# Aggressive Juvenile-Onset Respiratory Papillomatosis in a High HIV Prevalence Setting: Clinical Predictors of Severity in South Africa

**DOI:** 10.1093/ofid/ofaf741

**Published:** 2025-12-15

**Authors:** L A Sibiya, T Abel, S Maistry, R Seedat, J Z Porterfield, Y Liang, E Evangelista, M Tyle, Y Saman, N Msomi

**Affiliations:** Department of Otorhinolaryngology, Inkosi Albert Luthuli Central Hospital, Durban, KwaZulu-Natal, South Africa; Department of Otorhinolaryngology, School of Medicine, College of Health Sciences, University of KwaZulu-Natal, Durban, KwaZulu-Natal, South Africa; Department of Otorhinolaryngology, Inkosi Albert Luthuli Central Hospital, Durban, KwaZulu-Natal, South Africa; Department of Otorhinolaryngology, School of Medicine, College of Health Sciences, University of KwaZulu-Natal, Durban, KwaZulu-Natal, South Africa; Department of Otorhinolaryngology, Inkosi Albert Luthuli Central Hospital, Durban, KwaZulu-Natal, South Africa; Department of Otorhinolaryngology, School of Medicine, College of Health Sciences, University of KwaZulu-Natal, Durban, KwaZulu-Natal, South Africa; Department of Otorhinolaryngology, University of the Free State, Bloemfontein, Free State, South Africa; Division of Infectious Disease and International Medicine, College of Medicine, College of Public Health, University of South Florida, Tampa, Florida, USA; Division of Infectious Disease and International Medicine, College of Medicine, College of Public Health, University of South Florida, Tampa, Florida, USA; Division of Infectious Disease and International Medicine, College of Medicine, College of Public Health, University of South Florida, Tampa, Florida, USA; Division of Infectious Disease and International Medicine, College of Medicine, College of Public Health, University of South Florida, Tampa, Florida, USA; Department of Otorhinolaryngology, School of Medicine, College of Health Sciences, University of KwaZulu-Natal, Durban, KwaZulu-Natal, South Africa; Department of Brain Sciences, Imperial College London, London, UK; Discipline of Virology, School of Health Sciences, College of Health Sciences, University of KwaZulu-Natal, Durban, KwaZulu-Natal, South Africa; National Health Laboratory Service, Inkosi Albert Luthuli Central Hospital, Durban, KwaZulu-Natal, South Africa

**Keywords:** aggressive disease, human papillomavirus, juvenile-onset recurrent respiratory papillomatosis, KwaZulu-Natal, maternal HIV

## Abstract

**Background:**

Juvenile-onset recurrent respiratory papillomatosis (JoRRP) is a chronic, HPV-driven condition marked by recurrent airway papillomas. This study aimed to determine the prevalence and incidence of JoRRP and to identify clinical predictors of aggressive JoRRP.

**Methods:**

We conducted a retrospective analysis of JoRRP patients treated at Inkosi Albert Luthuli Central Hospital from 2012 to mid-2023. Demographics, patient HIV status, exposure to maternal HIV, frequency of surgical interventions, and extralaryngeal involvement were recorded.

**Results:**

The cohort of 277 patients had a median diagnosis age of 4 years. The incidence of JoRRP was 3.82 per 100 000 live births (95% CI, 2.86–5.01), and prevalence was 4.17 per 100 000 population (95% CI, 3.47–4.97). Half of the study cohort met the criteria for aggressive disease (AD) (139; 50%). Children diagnosed at ≤2 years of age had higher odds of AD than older children, 3–5 years (OR: 0.43, 95% CI: 0.24–0.78) and >5 years (OR: 0.30, 95% CI: 0.16–0.54); both *P* < .001. Additionally, exposure to maternal HIV was significantly associated with pulmonary involvement (*P* = .03).

**Conclusions:**

Early age at diagnosis and exposure to maternal HIV are potential predictors of aggressive JoRRP in high HIV-prevalence settings. These findings underscore the importance of integrated maternal–child healthcare, and robust public health interventions, such as expanded HPV vaccination and enhanced HIV prevention strategies, to reduce the clinical burden of JoRRP.

## Teaser

277 JoRRP cases; 50% aggressive. Onset at ≤2 years raises the risk of aggressive disease. Exposure to maternal HIV was associated with pulmonary spread. JoRRP incidence was 3.82 per 100 000 births and was prevalence 4.17 per 100 000 population (public-sector–adjusted). Prioritize early detection, HPV vaccination, HIV prevention.

Juvenile-onset Recurrent Respiratory Papillomatosis (JoRRP) is a rare but clinically significant disease characterized by the recurrent growth of papilloma within the respiratory tract [1]. It is a chronic, vaccine-preventable pediatric disease caused predominantly by human papillomavirus (HPV) types 6 and 11, typically acquired intrapartum [2]. While JoRRP is considered rare in high-income countries (HICs), emerging evidence from low and middle-income countries (LMICs), particularly in South Africa (SA), suggests a relatively higher incidence and prevalence [3, 4]. Globally, the incidence of JoRRP ranges from approximately 0.5 to 1.5 per 100 000 children, although regional estimates fall toward the upper end of this spectrum [3].

Transmission of HPV leading to JoRRP primarily occurs via vertical transmission from mother to child during vaginal delivery [5]. A maternal history of genital warts or active HPV infection heightens the risk of transmission [5]. Epidemiological studies have identified several risk factors associated with the development of JoRRP, including maternal age under 20 years, firstborn status, and prolonged labor [6]. Although cesarean section can reduce the risk of transmission, it does not eliminate it entirely [5].

Clinically, JoRRP manifests with the development of benign papillomatous lesions within the airway, predominantly affecting the larynx [1]. The hallmark initial symptom is progressive hoarseness or dysphonia, due to involvement of the vocal cords [7]. As the disease progresses, patients may experience stridor, chronic cough, and respiratory distress resulting from airway obstruction [8]. These symptoms typically present in early childhood, often before the age of 5 years, and can vary widely in severity among affected individuals [9]. Early recognition of these clinical features is essential for prompt diagnosis and intervention, which may reduce morbidity.

Aggressive disease (AD), also known as severe RRP, is marked by rapid papilloma proliferation and frequent recurrences that often require more than three surgical interventions in a 12-month period, increasing the risk of spread to the lower airways (trachea, bronchi, and lung parenchyma) [1, 10]. This aggressive progression can result in significant airway obstruction, higher morbidity, and, in about 1% of cases, malignant transformation into squamous cell carcinoma [11, 12]. Factors linked to AD include early age of onset, HPV type 11 infection, and specific immunological factors [1]. Recognizing these predictive clinical features is essential for early, tailored management, particularly in settings with high HIV prevalence and limited resources [10, 13].

KwaZulu-Natal (KZN) is a critical hotspot in SA, where nearly 22% of adults aged ≥15 years are with HIV and pediatric infections remain concerningly high [14]. Despite robust efforts to curb mother-to-child transmission, 2.4% of children aged 0–14 years live with HIV [15]. The combined effects of high maternal HIV rates, limited healthcare access, and great socioeconomic disparities drive these persistent transmission rates [16]. These striking statistics not only highlight a public health crisis in KZN but also offer a powerful lens through which to examine JoRRP amid widespread HIV which is a challenge echoing across similar resource-limited settings globally.

The prevalence of HPV infection in children in KZN is less well-documented compared with adult populations. However, studies indicate that HPV infection rates are higher in people with HIV (PWHIV) due to immunosuppression, which may extend to the paediatric population [17]. In regions like KZN, where both HIV and HPV are endemic, co-infection rates may be higher, although precise prevalence data in children are limited [18]. Research focusing on oropharyngeal and anogenital HPV infections in children with HIV suggests a higher susceptibility and persistence of HPV infections in this group [19].

Despite growing knowledge of JoRRP, key epidemiologic and severity drivers remain incompletely characterized in high-HIV regions such as KZN. Our study therefore characterizes the clinical factors that predict aggressive JoRRP in this setting, aiming to generate evidence applicable to other resource-limited, HIV-burdened areas. Clarifying these predictors will guide early triage, inform targeted public-health strategies, and help allocate scarce surgical and vaccination resources more effectively.

## METHODOLOGY

### Study Design

We conducted a retrospective chart review between 2012 and mid-2023 (11.5 years) at KZN's provincial quaternary referral centre, Inkosi Albert Luthuli Central Hospital, to identify demographic and clinical predictors of aggressive JoRRP in this high-HIV setting.

### Study Population

We analyzed JoRRP cases (≤12 years at time of diagnosis) that required surgery for airway patency during the 10-year period; only histologically confirmed patients with complete records were included, while non-surgical cases or those diagnosed after the age of 12 years were excluded.

### Data Collection

The demographic data collected included age at diagnosis, and sex. Clinical variables were gathered such as the presence of extralaryngeal disease, the frequency of surgical interventions, HIV status and whether patients were receiving combined antiretroviral therapy (cART). Maternal HIV status was also documented so that we could classify each child's perinatal exposure. Children who themselves tested HIV-negative but were born to an HIV-positive mother were labeled HIV-exposed, whereas those born to an HIV-negative mother were considered HIV-unexposed. All data from electronic patient files were extracted and entered into a Microsoft Excel spreadsheet which was password protected.

### AD Criteria

Patients were classified as having AD using previously defined criteria of meeting at least one of the following: (1) underwent at least ten surgeries over their lifetime; (2) required more than three surgeries in a 12-month period; (3) exhibited extralaryngeal disease; or (4) required a tracheostomy [20].

### Provincial Burden Estimates

*Design & window:* Retrospective chart review of all confirmed JoRRP cases in KZN among children born 2012–2019, aligning with the start of systematic capture (2012) and allowing adequate follow-up to the 2022 data close. Median age at diagnosis (∼4 years) and service transition by 10–14 years support this window (eg, 2012 births were ∼10 years old in 2022).

*Incidence (birth-cohort):* Pooled 2012–2019 births. Numerator: children born in 2012–2019 with any JoRRP encounter (ICD 14.1) in 2022. Denominator: sum of KZN live births 2012–2019 = 1 359 890. Reported per 100 000 births; no sub-banding (aimed to estimate absolute incidence, not time trends) [21].

*Prevalence (2022):* Numerator: all JoRRP patients with any encounter in 2022 within KZN. Denominator: KZN mid-year 0–14 y population in 2022 = 3 588 143. Reported per 100 000 children.

*Coverage adjustment:* Incidence and prevalence were adjusted for public-sector capture using coverage fraction c = 0.842 [22].

A detailed outline of the methodology used can be seen in Supplementary Appendix A.

### Statistical Analysis

Descriptive statistics were used to summarize the demographic and clinical characteristics of the patient cohort. Continuous variables were expressed as means with standard deviations or medians and interquartile ranges (IQR), while categorical variables were presented as frequencies and percentages. Logistic regression analysis was performed to identify factors associated with AD progression. We modeled AD using multivariable logistic regression. Age at diagnosis and HIV status were prespecified covariates and retained in all models. Other variables were examined in univariable analyses and included in multivariable models where clinically justified. Adjusted odds ratios (aORs) with 95% confidence intervals (CIs) are reported; two-sided *P* < .05 defined statistical significance. Exact Poisson 95% CIs were used for all rate estimates, treating case counts as Poisson and scaling by the appropriate denominator. ORs with 95% CIs were calculated to estimate the strength of the association between various factors and the likelihood of aggressive JoRRP progression. All statistical analysis was conducted using SPSS version 11. Statistical significance was set at a *P* value of <.05.

### Patient Consent Statement

This study was a retrospective chart review of existing ENT patient records. The Biomedical Research Ethics Committee (BREC) of the University of KZN approved the protocol and granted a waiver of individual informed consent for review of patient files (BREC Ref: BREC/00002826/2024). All data were de-identified prior to analysis and handled in accordance with institutional policies.

## RESULTS

### Patient Demographics

During the study period, a total of 277 patients were included, comprising 155 males (56%) and 122 females (44%) (Table 1). The median age at diagnosis was 4 years (IQR:6–2 years). With respect to HIV status, 17 patients (6%) were with HIV, and HIV status was unknown for 4 patients (1%). Patient cART treatment was reported in 17 patients (100%).

**Table 1.** Demographic and Clinical Characteristics

Variable	*N* (%)
Patient demographics	
Age at diagnosis, *median years (IQR)*	4 (2–6)
Gender	
Male	155 (56)
Female	122 (44)
HIV status	
HIV-positive	17 (6)
HIV-negative	256 (92)
Unknown	4 (1)
Patient HIV treatment^a^	
Receiving cART^b^	17 (100)
Not receiving cART^b^	0 (0)
Unknown	0 (0)
Exposure to maternal HIV	
HIV exposed	53 (34)^a^
HIV unexposed	101 (66)^a^
Unknown	123 (44)
Maternal HIV treatment^a^	
Receiving cART^b^	16 (30)
Not receiving cART^b^	5(9)
Unknown	32 (60)
Disease demographics	
Total surgeries within study period, *median (IQR)*	5 (12–2)
Pulmonary disease	24 (9)
Extralaryngeal disease	71 (26)
Nasopharyngeal	12 (4)
Oropharyngeal	15 (5)
Tracheal	54 (20)

^a^The denominator includes number of patients that HIV status was available for; ^b^cART, Combined antiretroviral therapy.

Exposure to maternal HIV status was known for 154 patients (56%); among these, 53 patients (34%) were HIV-exposed, and 101 patients (66%) were HIV-unexposed. The exposure status was unknown for 123 patients (44%). Maternal cART treatment was reported in 16 cases (30%), 5 mothers (9%) were not on treatment, and unknown in 32 cases (60%).

### Provincial Burden (2012–2022)

In KZN in 2022, the incidence rate of JoRRP was 3.82 per 100 000 births (95%CI: 2.86–5.01), based on 52 incident cases from the 2012–2019 birth cohorts with any JoRRP encounter in 2022. The prevalence for the same year was 4.17 per 100 000 children (95%CI: 3.47–4.97), based on 126 children active in care in 2022.

### Disease Characteristics

**Figure 1.**
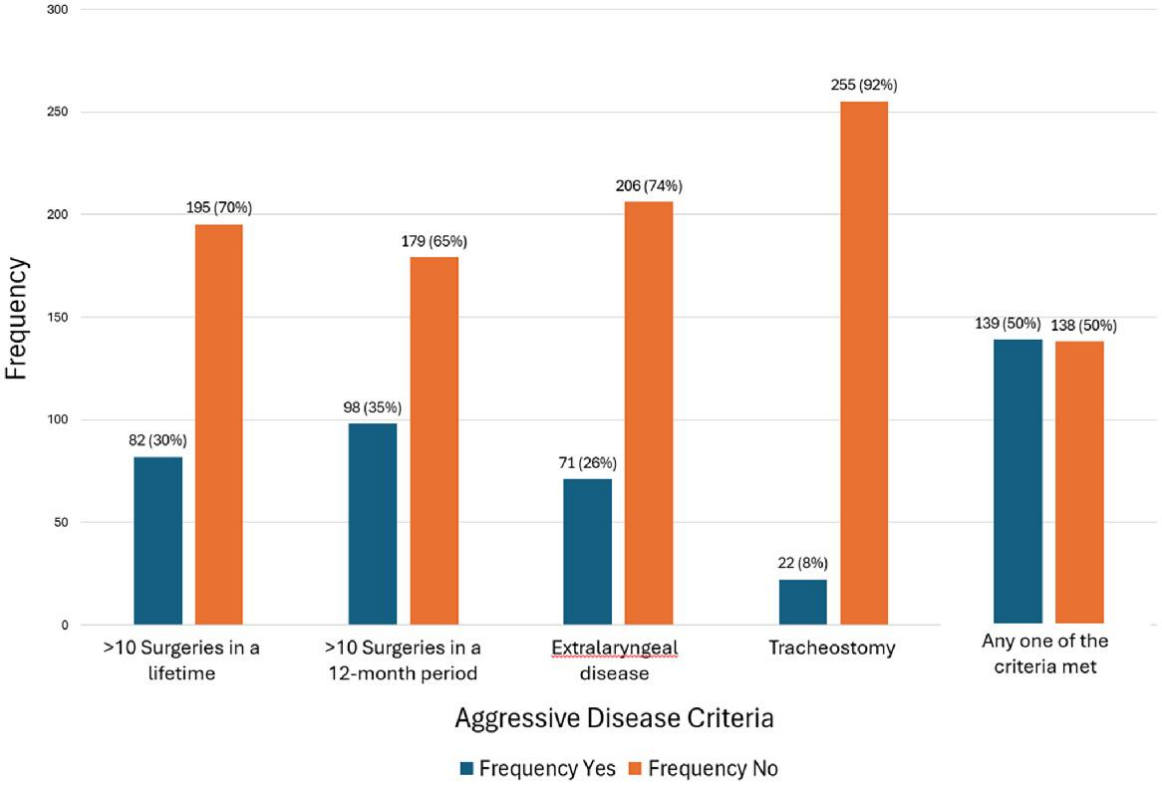
Bar chart showing the frequency of patients meeting or not meeting established aggressive disease criteria for juvenile-onset recurrent respiratory papillomatosis (JoRRP). Criteria included >10 surgeries in a lifetime, >3 surgeries within a 12-month period, presence of extralaryngeal disease, and history of tracheostomy. The final category indicates the proportion of patients who met at least one of these criteria which is the minimum requirement for having aggressive disease.

Within the cohort of 277 patients we found a median of 5 (IQR:12–2) total surgeries per patient over the 11-year study period. The criteria for AD were met by 139 patients (50%) which is represented in Figure 1. The commonest criteria met were more than three surgeries in a 12-month period (98 patients, 35%) and at least ten surgeries over a lifetime (82 patients, 29%).

Extralaryngeal disease was present in 71 patients (26%). Specific sites of involvement included pulmonary disease in 24 patients (9%) and nasopharyngeal involvement in 12 patients (4%). Tracheostomies were required in 22 patients (8%).

Our results indicate that age at onset significantly differs between patients with non-AD and AD (Table 2). Children diagnosed between ages 3–5 or older than 5 were significantly less likely to have AD compared with those diagnosed at the age of ≤2 years (OR:0.43 and 0.30, respectively; *P* < .001). Gender was not associated with AD. Regarding immunological parameters, children with a CD4 + count <200 were less likely to have AD compared with those with CD4 + counts >500, although the results did not reach statistical significance (adjusted OR:0.51; 95%CI: 0.05–5.82; *P* = .59). Similarly, viral load levels at baseline did not significantly differ between groups, although patients with HIV viral loads <1000 showed a trend toward lower odds of having AD compared with those with viral loads >1000 (adjusted OR:0.58; 95%CI: 0.17–2.00; *P* = .38).

**Table 2.** Factors Associated with Aggressive Disease

Demographic Factor	Category	Non-AD (n = 138) n (%)	AD (n = 139) n (%)	Unadjusted OR (95% CI)	*P V*alue	^a^Adjusted OR (95% CI)	*P V*alue
Age at onset	≤2 (Reference)	36 (26)	69 (50)	ref	-		
	3–5	46 (33)	38 (27)	0.43 (0.24–0.78)	<.001		
	>5	56 (41)	32 (23)	0.30 (0.16–0.54)	<.001		
Gender	Male (Reference)	82 (59)	73 (53)	ref	-		
	Female	56 (41)	66 (48)	1.32 (0.82–2.13)	.25	1.41 (0.85–2.34)	.18
HIV status	Negative (Reference)	129 (94)	126 (91)	ref	-		
	Positive	6 (4)	11 (9)	2.05 (0.75–5.62)	.16		
	Unknown	3 (2)	1 (1)	Excluded			
Exposure to maternal HIV	Not Exposed (Reference)	50 (36)	51 (37)	ref	-		
	Exposed	24 (17)	29 (21)	1.18 (0.62–2.38)	.62	1.16 (0.55–2.45)	.70
	Unknown	64 (46)	59 (42)	Excluded			
CD4+ count (cells/mm3)^b^	<200	0 (0)	1 (1)	0.38 (0.03–5.40)	.48	0.51 (0.05–5.82)	.59
	201–500	0 (0)	0 (0)				
	>500 (Reference)	6 (4)	10 (7)	ref	-		
	Unknown	0 (0)	(0)	Excluded			
HIV viral load at baseline (copies/ml)^b^	<1000	0 (0)	4 (3)	0.36 (0.08–1.71)	.20	0.58 (0.17–2.00)	.38
	>1000 (Reference)	2 (1)	1 (1)	ref	-		
	Undetectable	4 (3)	6 (4)	Excluded			
	Unknown	0 (0)	0 (0)	Excluded			

^a^Adjusted for age at diagnosis and HIV status.

^b^Total number of HIV-positive patients were 17.

Pulmonary involvement was compared across various demographic factors. The age at onset, gender, and HIV status did not show significant differences in relation to pulmonary involvement. However, exposure to maternal HIV was notably significant (*P* = .03), suggesting a potential link between exposure to maternal HIV and pulmonary involvement (Table 3). Importantly, 116 patients had missing HIV exposure records.

**Table 3.** Association of Demographic Factors with Pulmonary Disease

Demographic factor	Category	No Pulmonary Disease (n = 253) n (%)	Pulmonary Disease (n = 24) n (%)	*P V*alue
Age at onset	≤2	92 (36)	13 (54)	.22
	3–5	79 (31)	5 (21)	
	>5	82 (32)	6 (25)	
Gender	Male (Reference)	144 (57)	11 (46)	.58
	Female	109 (43)	13 (54)	
HIV status	Neg (Reference)	232 (92)	23 (96)	.81
	Pos	17 (7)	1 (4)	
	Unknown	3 (1)	1 (4)	
Exposure to maternal HIV	Not Exposed (Reference)	94 (37)	7 (29)	.03
	Exposed	43 (17)	10 (42)	
	Unknown	116 (46)	7 (29)	

No significant differences were observed in the age at onset, gender, or HIV status between patients with and without extralaryngeal involvement (Table 4). However, those diagnosed at the age of ≤2 years showed a higher proportion of extralaryngeal involvement compared with other age groups.

## DISCUSSION

### Summary of Key Findings

This study presents one of the largest cohorts (n = 277) of JoRRP patients reported from a single institution. By analyzing and correlating important clinical and demographic aspects of these patients, we aimed to understand their association with AD in KZN, SA which is a high HIV-prevalence region [15]. In KZN in 2022, the incidence rate of JoRRP was 3.82 per 100 000 births. The prevalence for the same year was 4.17 per 100 000 children. Importantly, half of our study population (50.2%) met the criteria for AD, emphasizing the substantial disease burden of JoRRP in this region (Figure 1).

**Figure 2.**
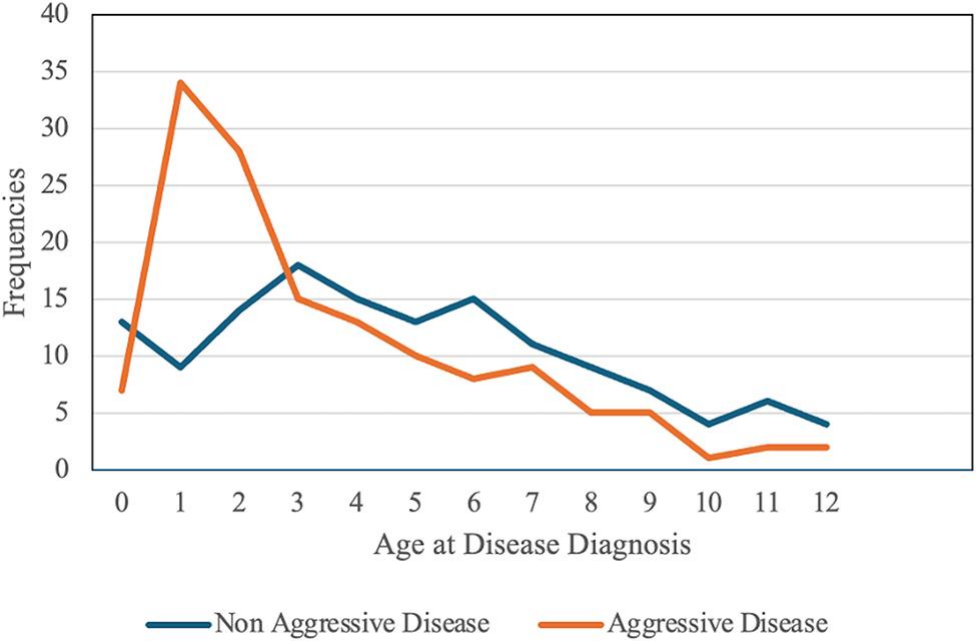
Age at disease diagnosis. Age at disease diagnosis among patients with juvenile-onset recurrent respiratory papillomatosis (JoRRP). The blue line represents patients without meeting aggressive disease criteria, while the orange line represents patients with aggressive disease.

Our findings indicate that patients who presented at ≤2 years of age were significantly more likely to develop AD compared with those who presented at older ages (Figure 2). Specifically, compared with patients presenting at ≤2 years, those presenting at ages 3–5 years had a significantly lower likelihood of developing AD, and those presenting at >5 years had a further reduced likelihood (Table 2). Gender was not significantly associated with AD. Patients with AD exhibited extensive laryngeal involvement.

Patient HIV status revealed a clear trend toward an increased likelihood of AD, with PWHIV showing higher odds of the disease. Furthermore, exposure to maternal HIV emerged as a statistically significant factor, particularly regarding pulmonary involvement. Patients born to mothers with HIV demonstrated a markedly higher incidence of pulmonary complications compared with those born to HIV-negative mothers (Table 3). In our study population, nearly 9% experienced pulmonary complications stemming from JoRRP.

### Interpretation of Findings

**Figure 3.**
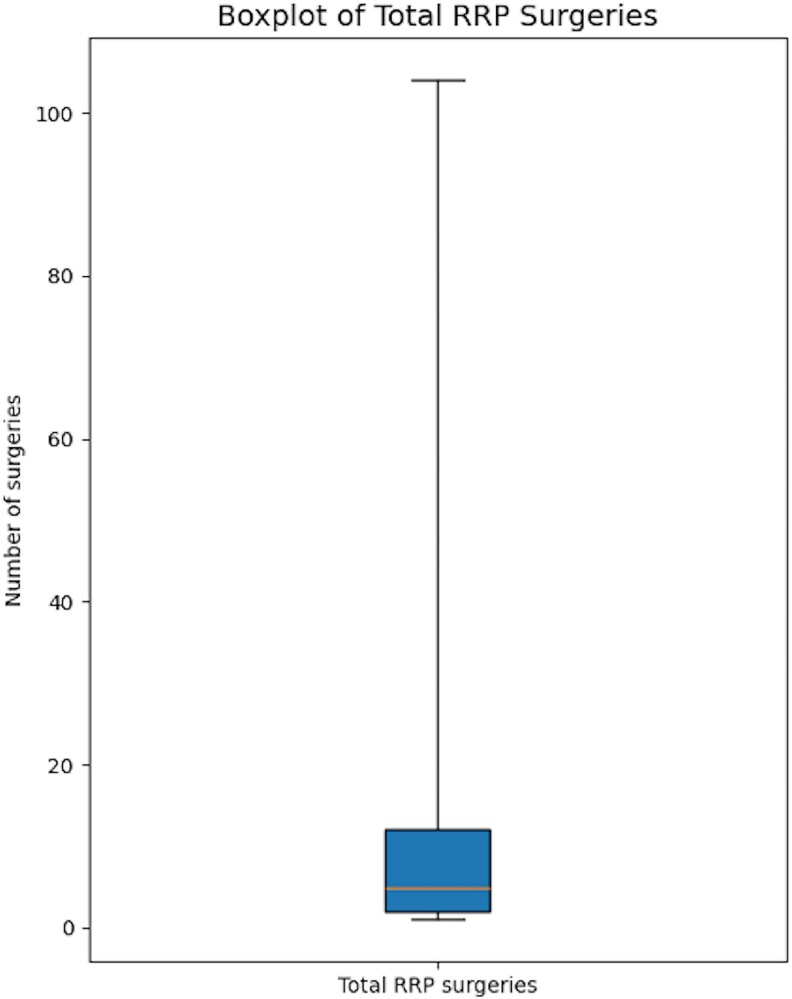
Box-and-whisker plot of total surgeries among patients with juvenile-onset recurrent respiratory papillomatosis (JoRRP). The box spans the interquartile range (Q1–Q3), the line inside the box represents the median, and whiskers extend to the minimum and maximum observed values.

**Figure 4.**
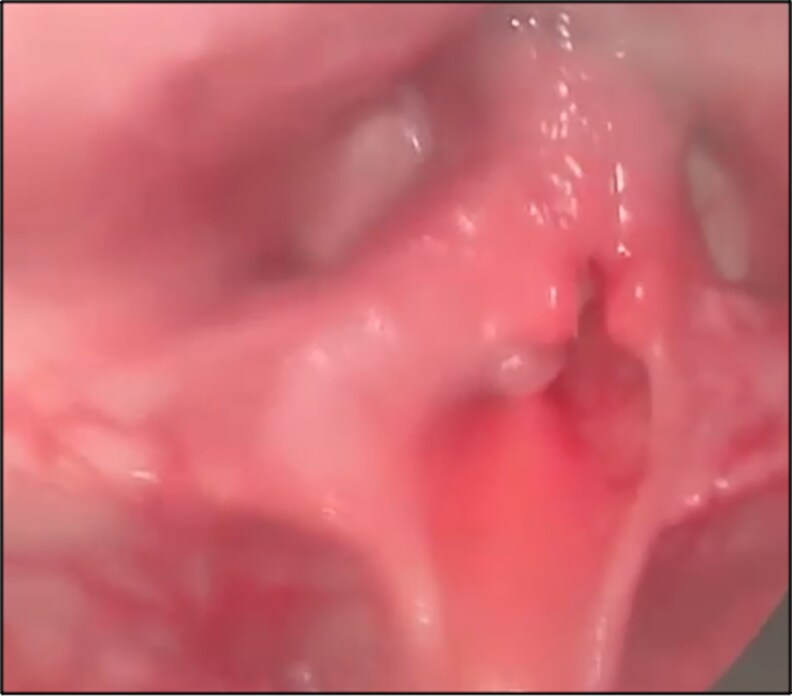
Endoscopic laryngeal evaluation of a patient with JoRRP. This patient presented with progressive hoarseness, stridor, and respiratory distress, indicative of airway obstruction. The endoscopic examination revealed papillomatous lesions extensively involving the vocal cords and laryngeal structures. The lesions, characteristic of JoRRP, were surgically excised to restore airway patency.

Our study demonstrates a strong association between early age at onset and the development of AD in JoRRP patients, with 50.2% of our cohort exhibiting AD. The rates of AD observed in our study are supported by published literature albeit, they are multicentre studies [23]. Patients presenting at ≤2 years of age were significantly more likely to develop AD compared with those presenting at ages 3–5 years and >5 years, supported by earlier literature [24]. This finding underscores the critical role of immunological maturity and anatomical factors in disease progression [25, 26]. Younger children's immature immune systems may limit their ability to control HPV infections. If they acquire a larger initial viral inoculum, influenced by both virological and host factors, it is plausible that these factors could contribute to earlier presentation and more AD. Additionally, smaller airway sizes in younger patients may exacerbate disease severity, complicate surgical interventions, and promote frequent surgical interventions to prevent airway obstruction [24]. The cumulative surgical burden was highly right-skewed, with a small subset requiring dozens of procedures (Figure 3), underscoring the intensity of care among the most severely affected children. Figure 4 illustrates exophytic papillomas narrowing the glottis, a visual correlate of the high surgical burden shown in Figure 3.

In our provincial cohort, the incidence was 3.82 per 100 000 births, and the 2022 prevalence (0–14 y, encounter-based) was 4.17 per 100 000 children. These estimates are higher than several international reports and local data from SA. In the Free State (2011–2013), Seedat reported incidence 1.34 per 100 000 population and prevalence 3.88 per 100 000; Lesotho estimates were 0.49 and 1.04 per 100 000, respectively [4]. Our figures also sit above US city-based estimates from Atlanta and Seattle (incidence 0.36–1.11; prevalence 1.69–2.59 per 100 000 children) and Canada's national registry (incidence 0.24; prevalence 1.11 per 100 000 children) [9, 27]. By contrast, post-vaccine US birth-cohort surveillance documented declines from 2.0 cases in 2004–2005 to 0.5 cases in 2012–2013 [21]. Together, these comparisons are consistent with heterogeneity by method (births vs child-population denominators; encounter-based prevalence), health-system context, and HPV vaccination timelines and coverage. This study estimated incidence using a birth-cohort framework with live-birth denominators; because many prior reports used child-population denominators, registries, or cross-sectional designs, direct rate comparisons are not appropriate. Gender was not significantly associated with AD. These observations align with global studies that report an even gender distribution in JoRRP severity [28].

Our study revealed that PWHIV had an increased likelihood of developing AD, with an OR of 2.05 (*P* = .16), suggesting a potential association between HIV status and AD risk. Among the 17 patients for whom viral load data were available, 11 had undetectable viral loads, highlighting the effectiveness of cART in this cohort. The high rate of viral suppression may have mitigated the impact of HIV on disease progression, potentially influencing the observed association [29].

We found a significant association between exposure to maternal HIV and pulmonary involvement (*P* = .03). Patients born to mothers with HIV had a higher incidence of pulmonary complications compared with those born to HIV-negative mothers (Table 4). Maternal HIV infection may affect the neonatal immune system through mechanisms such as in utero exposure to the virus, immune complexes, or altered cytokine environments [25]. These factors could impair the child's innate and adaptive immune responses, increasing susceptibility to severe HPV-related diseases like JoRRP. Global studies report pulmonary involvement in JoRRP in only 3%–9% of cases [30–32]; notably, our cohort reaches the upper limit at 9%, underscoring an exceptionally high prevalence of pulmonary complications.

**Table 4.** Association of Demographic Factors with Extralaryngeal Disease

Demographic Factor	Category	No Extralaryngeal Disease (n = 206) n (%)	Extralaryngeal Disease (n = 71) n (%)	*P V*alue
Age at onset	≤2	71 (34)	34 (48)	.12
	3–5	67 (33)	17 (24)	
	>5	68 (33)	20 (28)	
Gender	Male (Reference)	114 (55)	41 (58)	.72
	Female	92 (45)	30 (42)	
HIV status	Neg (Reference)	193 (94)	62 (87)	.09
	Pos	10 (5)	8 (11)	
	Unknown	3 (1)	1 (1)	
Exposure to maternal HIV	Not Exposed (Reference)	71 (34)	30 (42)	.35
	Exposed	41 (20)	12 (17)	
	Unknown	94 (46)	29 (41)	

Nasopharyngeal involvement was assessed across different demographic variables (Table 5). There were no significant associations identified.

**Table 5.** Association of Demographic Factors with Nasopharyngeal Disease

Demographic Factor	Category	No Nasopharyngeal Disease (n = 265) n (%)	Nasopharyngeal Disease (n = 12) n (%)	*P V*alue
Age at onset	≤2	99 (37)	6 (50)	.46
	3–5	80 (30)	4 (33)	
	>5	86 (32)	2 (17)	
Gender	Male (Reference)	147 (55)	8 (67)	.45
	Female	118 (45)	4 (33)	
HIV status	Neg (Reference)	244 (92)	11 (92)	.9
	Pos	18 (7)	0 (0)	
	Unknown	3 (1)	1 (8)	
Exposure to maternal HIV	Not Exposed (Reference)	96 (36)	5 (42)	.6
	Exposed	51 (19)	2 (17)	
	Unknown	118 (45)	5 (42)	

In-utero exposure to maternal HIV antigens and antiretroviral therapy can re-program innate and adaptive immune responses in HIV-exposed uninfected infants, leading to altered cytokine profiles, impaired dendritic-cell function, and reduced virus-specific T-cell responses [25, 29]. These immune alterations may diminish a child's capacity to control early HPV infection, accelerating papilloma proliferation and surgical burden. Importantly, our data suggest that even when vertical HIV transmission is prevented, perinatal immune imprinting may still influence the trajectory of other viral diseases such as JoRRP, reinforcing the need for intensified surveillance and anticipatory counseling for HIV-exposed uninfected children in high-HIV-prevalence settings. These findings are consistent with an association between perinatal HIV exposure and greater disease severity, with implications for maternal–child health policy in high-HIV settings.

Our analysis revealed no significant differences in CD4 counts and viral loads between PWHIV with non-AD and AD, although the limited sample size (n = 17) might have obscured subtle disparities [29]. This indicates that overall immunosuppression, as measured by these parameters, may not be the main driver of AD; instead, localized immune responses or HPV-specific immunity could play a more critical role in disease progression [26].

HIV infection can undermine the effectiveness of HPV prophylaxis, as PWHIV may exhibit a reduced immunogenic response to the HPV vaccine, diminishing its protective benefits [33]. Yet, early initiation of cART in HIV-infected children can boost immune function and enhance the vaccine response [34]. This underscores the critical need for integrated healthcare strategies that simultaneously address HIV management and HPV prevention, a globally relevant imperative as international efforts continue to expand HPV vaccination and reduce maternal–child HIV transmission.

This study found that almost 9% of our population had pulmonary complications arising from JoRRP. Pulmonary involvement is a severe manifestation of JoRRP and is associated with significant morbidity and mortality due to the risk of airway obstruction and potential malignant transformation [11]. The significant association between exposure to maternal HIV and pulmonary involvement underscores the importance of maternal health in the disease severity of JoRRP.

### Public Health Implications

Vaccination against HPV is effective in preventing JoRRP. However, this benefit is not fully realized in practice, potentially due to outdated National Vaccination Programs that do not cover all relevant HPV types. The current state-funded vaccination program in South Africa includes the bivalent vaccine that covers HPV types 16 and 18, although the quadrivalent and nonavalent vaccines are available in the private sector. HPV vaccines, which include HPV types 6 and 11, commonly associated with RRP, have shown promise, with near-elimination of new JoRRP cases in Australia [35]. Failure to use HPV vaccines that include HPV types 6 and 11 may represent a missed opportunity to prevent RRP [3]. Challenges such as vaccine accessibility, cost, and public awareness must be addressed to improve uptake, particularly in resource-limited settings like KZN [3, 36]. Furthermore, targeted vaccination campaigns for post-exposed, sexually debuted adults, especially PWHIV who were not included in the national HPV program when they were in school, should be considered as an innovative strategy. Due to JoRRP being caused predominantly by HPV types 6 and 11, a bivalent (16/18) program will not meaningfully reduce JoRRP incidence. Prevention requires use of vaccines that include HPV types 6 and 11. Transitioning the public program to a 6- and 11-containing product (with targeted catch-up for unvaccinated cohorts) is therefore the key policy lever to reduce JoRRP and its surgical burden. Exploring the immune response to vaccination after exposure may potentially reduce their risk of developing HPV-related conditions and lower transmission risk to their children.

The significant association between exposure to maternal HIV and pulmonary involvement suggests that strengthening PMTCT (Prevention of Mother to Child Transmission) programs could have implications for reducing the severity of JoRRP in children. Importantly, our cohort had a substantial number of unknowns for maternal HIV exposure. Ensuring that pregnant women have access to HIV testing and ART could reduce in utero exposure to HIV and potentially mitigate the risk of developing severe JoRRP [37]. These findings reinforce the need for integrated infectious disease control strategies; specifically, enhanced HPV vaccination and effective HIV management, to reduce both the incidence and severity of JoRRP.

JoRRP, though rare, highlights the far-reaching consequences of common viral infections (HPV) in children and the interplay with other infections like HIV. Lessons from our study support the paradigm that controlling maternal infections (be it HPV via vaccination or HIV via cART) can profoundly affect child health outcomes. This aligns with global infectious disease control goals and may serve as indicators of population-level infection control.

Additionally, although our study did not determine the local HPV genotype distribution, Free State data indicate ∼60% HPV-6 and ∼40% HPV-11 among RRP cases; in that cohort, HPV-11 JoRRP presented at a younger age than HPV-6 (median 3.2 vs 5.6 years), and HPV-11 has been linked to a more aggressive clinical course [38]. Given the high proportion of aggressive cases in our cohort and early local molecular-epidemiological signals, we hypothesise that HPV-11 is disproportionately common in KZN; patient-level genotyping will be needed to test this hypothesis.

### Limitations of the Study

The retrospective design of the study risks information bias from incomplete records. Small immunology subsamples among PWHIV (CD4 count and viral load: n = 17) reduce power. Missing maternal HPV status and the absence of HPV genotyping preclude assessing genotype–severity associations. As a single-centre study, generalizability may be limited. ART adherence/timing in mothers and children was not assessed. Key perinatal covariates, maternal age at delivery and birth order, were not captured. Finally, burden estimates may be biased by the assumption of a fixed 84.2% public-sector fraction.

### Future Research Directions

Prospective studies incorporating HPV genotyping are needed to elucidate the role of specific HPV types in the aggressiveness of JoRRP. Investigating the immunological profiles of patients, including cytokine levels, immune cell function, and mucosal immunity, elucidate biomarkers for AD [26]. Research into the mechanisms by which exposure to maternal HIV influences disease severity could inform targeted interventions aimed at mitigating this risk. Further studies examining the impact of adjuvant therapies and strategies to reduce surgical burden are warranted. Exploring the influence of maternal health, particularly HIV status and cART during pregnancy, on the severity of JoRRP in offspring could provide valuable insights for preventive strategies.

Moreover, future investigations should consider targeted vaccination campaigns for post-exposed, sexually debuted adults, particularly women with HIV who were not included in the national HPV program during their school years. Evaluating the immune response to vaccination after exposure in this cohort could offer an innovative strategy to reduce their risk of HPV-related conditions and limit transmission to their children, recognizing that widespread rollout in LMICs may be challenging.

## CONCLUSION

Our study shows that an early age of onset and exposure to maternal HIV are significant predictors of aggressive JoRRP in KZN, SA. While overall HIV status did not correlate with disease aggressiveness, children exposed to maternal HIV experienced higher rates of pulmonary involvement, highlighting the critical role of maternal health. The high number of surgeries and lung complications demand prompt diagnosis, integrated maternal and child healthcare, and targeted adjuvant therapies. Our findings emphasize that comprehensive public health strategies, including quadrivalent HPV vaccination for both boys and girls and robust HIV prevention and management, may help reduce the incidence and severity of this vaccine-preventable disease in high-HIV prevalence and resource-limited settings.

## Supplementary Material

ofaf741_Supplementary_Data

## Supplementary Data

Supplementary materials are available at *Open Forum Infectious Diseases* online. Consisting of data provided by the authors to benefit the reader, the posted materials are not copyedited and are the sole responsibility of the authors, so questions or comments should be addressed to the corresponding author.
