# Supplementary material for: Aggressive Juvenile-Onset Respiratory Papillomatosis in a High HIV Prevalence Setting: Clinical Predictors of Severity in South Africa
Source: Open Forum Infect Dis. 2025 Dec 15;13(1):ofaf741. doi: 10.1093/ofid/ofaf741 (PMC12750325; doi:10.1093/ofid/ofaf741)
Supplement: ofaf741_Supplementary_Data [file ofaf741_supplementary_data.docx]

**Supplementary Material**

**Supplementary A: Incidence and Prevalence Methodology**

To assess numbers of JoRRP case-patients over time under consistent surveillance, we conducted a retrospective chart review to identify all children with confirmed JoRRP who were born during 2012–2019 within the KZN province. This range of birth years was selected to align with the start of systematic data collection in 2012 and to ensure sufficient follow-up. The analytic period was therefore bounded by (i) the initiation of consistent case capture in 2012 and (ii) births up to 2019, ending 4 years before the most recent complete data retrieval date in 2022. This design ensured that later birth cohorts had accrued adequate opportunity for JoRRP diagnosis while limiting incomplete follow-up. In our setting, children generally transition out of otorhinolaryngology services from 10–14 years of age; by 2022, those born in 2012 were 10 years old, providing full capture for the earliest cohorts. This approach mirrors prior birth-cohort incidence studies that define the analytic window based on the cohort's median age at diagnosis (4 years in our population) and the availability of complete data [21].

To calculate incidence, we used denominators from Statistics SA statistics on live births in KZN. We pooled birth years 2012–2019: the numerator was the number of children born during 2012–2019 who had any JoRRP encounter (ICD 14.1) in 2022; the denominator was the sum of live births in KZN from 2012 through 2019 which was 1 359 890 babies. Rates are presented as cases per 100 000 births. Unlike previous reports that aggregated cases into 2-year birth intervals to compare early vs. late periods [21], we analyzed the entire 2012–2019 birth cohort as a whole without sub-banding, because our objective was estimation of absolute incidence rather than temporal comparison.

We estimated 2022 prevalence as follows: the numerator included all patients with JoRRP who had any JoRRP encounter in 2022 within the province; the denominator was the KZN mid‑year population aged 0–14 years in 2022, which was 3 588 143 children [22]. Prevalence is reported as cases per 100 000 children. Incidence and prevalence were adjusted for public-sector capture by dividing by the coverage fraction (c = 0.842) [22].
